# Prevalence of vitamin D deficiency and associated comorbidities among Abu Dhabi Emirates population

**DOI:** 10.1186/s13104-019-4536-1

**Published:** 2019-08-14

**Authors:** Amal Abdul Rahim Al Zarooni, Fatima Ibrahim Al Marzouqi, Salma Hamad Al Darmaki, Engela Adriana Margrietha Prinsloo, Nico Nagelkerke

**Affiliations:** 1 Ambulatory Healthcare Services, Seha, P.O. Box 19668, Al Ain, UAE; 2 Ministry of Health and Prevention, P.O. Box 679, Ajman, UAE; 3 Tawam Hospital, Seha, P.O. Box 15254, Al Ain, UAE; 4 Department of Family Medicine, College of Medicine and Health Sciences, United Arab Emirates University, P.O. Box 17666, Al Ain, UAE; 5 Community Medicine, College of Medicine and Health Sciences, United Arab Emirates University, Al Ain, UAE

**Keywords:** Vitamin D deficiency, Cardiovascular risk, Obesity, Hyperlipidemia, Impaired glucose, Diabetes mellitus

## Abstract

**Objective:**

The study explored the prevalence of vitamin D deficiency, its seasonal variation and associated comorbidities among the Abu Dhabi Emirati population living in urban and suburban settings.

**Result:**

Of the 12,346 participants 36.9% were male and 63.1% female. The majority (72%) were either vitamin D deficient (< 50 nmol/L), or (10%) vitamin D insufficient (50–74 nmol/L). Vitamin D deficiency was similar in both sexes (male 83.1% vs female 83.8%) as insufficiency (male 12.7% vs female 11.2%). Low vitamin D levels were associated with high blood pressure, high body mass index, central obesity, high cholesterol, impaired blood glucose levels and a high Framingham risk score. The mean vitamin D level was highest in January (winter) and lowest in July (summer).

## Introduction

Intestinal absorption of calcium and phosphate depends on the presence of Vitamin D, a fat soluble secosteroid, of which in humans the most important compounds are vitamin D2 (Ergocalciferol) and vitamin D3 (Cholecalciferol). Several studies reported a rapid growth of vitamin D deficiency among pediatric and adult populations globally in the past decade [1, 2]. A study published by Al-Othman et al. referred to 25(OH)D levels lower than 20 ng/ml in about 30% to 50% of children and adults, in the UAE, Australia, Turkey, India and Lebanon [1]. Low intake of vitamin D and calcium (confounded by lack of mandatory vitamin D milk supplement), and other factors such as, obesity, low social status, have been associated with low-level of vitamin D [1, 3–7].

A previous UAE study consisting of female participants only showed that the mean serum 25OHD concentrations were lowest in August and highest in April the time that marked the end of the short cooler winter season [8]. A study carried out amongst Saudi and expatriate participants reported counterintuitive findings of higher Vitamin D levels during winter compared to summer [9]. Other regional studies reported low Vitamin D levels among university students and patients visiting primary health care clinics [10, 11]. In a review carried out by Palomar, González-Clemente, Blanco-Vaca and Mauricio, it is revealed that Vitamin D deficiency alters insulin synthesis and secretion in both humans and animal models, which predisposes to glucose intolerance. They concluded that replenishing Vitamin D in patients with type 2 diabetes and established hypovitaminosis D improve glycemia and insulin secretion, thereby suggesting a role for vitamin D in the pathogenesis and management of type 2 diabetes mellitus [12]. However, in a recent randomized controlled trial (RCT) evidence to the contrary was reported [13].

Multiple epidemiological studies reported an association between vitamin D levels cardiovascular diseases, autoimmune diseases (with skeletal and extra skeletal involvement) and neoplastic diseases that included colon and breast cancer [10, 13–15]. With the regional study results in mind, we planned a study focusing on seasonal variation in a larger sample population of male and female participants in the UAE [1, 3, 7–11].

The present study was conducted to explore the prevalence and severity of vitamin D deficiency among the Abu Dhabi Emirati population investigating the seasonal relationship of vitamin D levels, habitat (urban, suburban) and comorbidities (diabetes mellitus, hypertension, hyperlipidemia, and obesity). In addition, the study explored the possible association between vitamin D deficiency, and cardiovascular risk as measured by the (Framingham risk score).

## Main text

### Methods

#### Subject

All adult participants ≥ 18 years of age, who presented at Ambulatory Health Care clinic for Weqaya Screening Program between Octobers 2011 and November 2012, were recruited. Written informed consent was obtained at the time of the Weqaya screening test. Weqaya (prevention) screening program is based on the Abu Dhabi Government screening program that identifies cardiovascular risk factors. The program measures (body mass index (BMI), blood pressure (BP), smoking status, HbA1c, total cholesterol/HDL ratio, vitamin D level, and creatinine level. The study protocol was approved by the Human Research Ethics Committee of Al Ain Medical District.

#### Study design and data collection

A cross sectional retrospective observational study was conducted. Data was collected through Weqaya register, and the report was generated from Cerner (Electronic medical records used by all Abu Dhabi heath facilities).

#### Sampling method

All adults ≥ 18 years of age (n = 12,513) who presented to Ambulatory Health Care centers for Weqaya Screening Program between October 2011–November 2012 were considered for the study After excluding participants with chronic kidney disease (stage III and IV), we included 12,346 participants in the study. We defined the cutoff value for vitamin D serum levels as follows: deficient (< 50 nmol/L), insufficient (50–74 nmol/L) and normal (≥ 75 nmol/L).

#### Inclusion and exclusion criteria

All adults ≥ 18 years of age, and United Arab Emirates Nationals who presented at Ambulatory Health Care centers for Weqaya Screening Program between October 2011 and November 2012, were included in the study. Patients suffering from chronic kidney disease (stage III and IV) were excluded.

#### Statistical analysis

Data were analyzed using the SPSS program version 19.

In addition to standard descriptive and basic inferential statistics such as the Chi square test for homogeneity in cross tabulations, and the independent samples t-test to compare mean values between groups, multiple linear regression was used to explore the determinants of vitamin d level.

### Results

A total of 12,346 participants were included in the study of which 63.1% were females and 36.9% were males. The age varied between 18 and 106 years with the mean of 38.5 years. Regarding the education level, most of the participants (47.8%) had high school education and the rest of the participants had a different level of education including PhD/master (1.6%), college/university (25.9%), and informal education (14.3%). Of the participants, 96.8% were from urban areas, while only 3.2% were from suburban areas.

About two-thirds of the participants were either obese (35.3%) or overweight (29.5%). Moreover, 17.4% of the participants had diabetes, while 23.7% was pre-diabetic. In addition, 14% of the participants had hypertension, and 30.2% had high cholesterol levels. Only in 7.8% of the participants reported to be smokers (Table 1**)**.

**Table 1:** General participant characteristics

	Character	Percentage	Number/12,346
Sex	Female	63.1	7785
	Male	36.9	4561
Age range (mean)	18–106 (38.5)		
Education level	PhD/master	1.6	202
	College/University	25.9	3199
	Primary School	47.8	5896
	No formal education	14.3	1770
Area	Urban	96.8	11,951
	Suburban	3.2	395
BMI	Obesity	35.3	4354
	Overweight	29.5	3636
	Normal	23.7	2924
	Underweight	3.1	378
Diabetic mellitus	Yes	17.4	2153
Pre diabetic	Yes	23.7	2929
Hypertension	Yes	14	1724
High cholesterol	Yes	30.2	3728
Smoking	Yes	7.8	965

#### Prevalence of vitamin D deficiency

The following cutoff values for vitamin D were defined: deficient (< 50 nmol/L), insufficient (50–74 nmol/L) and normal (≥ 75 nmol/L).

Based on this definition, vast majority (72%) were vitamin D deficient, 10% were vitamin D insufficient, and only 4.1% had normal vitamin D levels. Table 2 demonstrates the relationship between vitamin D levels and socio-demographic data. Participants younger than 30 years were vitamin D deficient more than those older than 30 years. In addition, participants with a higher level of education had lower vitamin D levels.

**Table 2:** Vitamin D level in relation to socio-demographic data

Socio-demographic data	Deficiency (%)	Insufficiency (%)	Normal (%)	P value
Age group				
< 30 years	93.8	4.7	1.5	.000
≥ 30 years	78.3	15.4	6.3	
Sex				
Female	83.8	11.2	5	.013
Male	83.1	12.7	4.2	
Education level^a^				
High	88.9	8.0	3.1	.000
Low	84.5	11.0	4.5	
No formal education	70.9	20.8	8.3	
Employment status				
Employed	88	9.1	2.9	.000
Housewife	78.7	15	6.3	
Retired	69.7	21.1	9.2	
Student	95.1	3.3	1.5	
Other	84.6	10.7	4.6	

^a^High education (PhD/master, college, university); low education (school); no formal education (never attended school)

The mean vitamin D levels were at its highest in January, February and April, which represented the short winter season. However, the mean vitamin D levels were at its lowest in July and August, which were the hottest months in the summer. The study showed no difference in mean vitamin D level concentration between the urban and suburban area.

#### Association of vitamin D with co-morbidities and cardiovascular risk

Low vitamin D was observed in participants who had high blood pressure (systolic and diastolic), high body mass index, central obesity, and high cholesterol (Table 3). The Framingham risk score (FRS) is used to estimate the 10-year cardiovascular risk of an individual. FRS calculation is based on age, gender, total cholesterol, HDL, systolic blood pressure, smoking and medication for hypertension. Our study showed an inverse relationship between vitamin D level and FRS consistent with our finding that most of the score components (BP, total cholesterol, and smoking) had an inverse relationship with vitamin D that HDL level had positive relation with vitamin D (Table 3). It was also revealed that high HbA1c level was associated with high levels of vitamin D, but impaired glucose tolerance was high in participants with vitamin D deficiency (Table 3).

**Table 3:** Multivariate linear regression model for plasma vitamin D level (dependent)

Independent variables	β2	P value	Standardized β	95.0% confidence interval
Age (years)	.406	.000	.609	(.573 to .645)
BMI (kg/m^2^)	− .018	.042	− .009	(− .018 to .000)
HBA1c %	.022	.029	.453	(.045 to .861)
Impaired glucose tolerance (IGT) mmol/L	− .031	.001	− 1.520	(− 2.394 to − .646)
Systolic blood pressure (SBP) (mmHg)	− .035	.007	− .050	(− .086 to − .014)
High density lipoprotein (HDL) mmol/L	.021	.033	1.260	(.102 to 2.417)
Total cholesterol mmol/L	− .165	.000	− 3.430	(− 3.812 to − 3.049)
Framingham risk score (FRS)	− .051	.000	− .264	(− .381 to − .147)

### Discussion

The results reveal an alarmingly high prevalence of vitamin D deficiency despite the fact that the participants live in the sunny Arabian Gulf region. The finding was not totally surprising because there were similar findings in the literature that focused on the region [1, 3, 7–11]. Multiple factors contribute to this finding, which includes sun exposure, atmospheric pollution, the degree of physical activity, clothing, cultural and dietary habits.

For seasonal variation, the results of our study were similar to the findings of a study carried out amongst Saudi local and expatriate participants, which reported unexpected higher Vitamin D levels during winter compared to summer [1, 9]. We studied the difference in vitamin D level between people living in urban and suburban areas to test the hypothesis that people living in suburban areas had higher vitamin D due to less atmospheric pollution and different lifestyles. However, the result did not support our hypothesis. This finding could be because people living in suburban areas changed their lifestyle similar to those living in urban areas. The vast difference in sample size between suburban and urban areas (3.2% vs 96.8%), could also be a reason for the finding, however, a larger suburban sample should be tested.

Worstman et al. reported a relation between high BMI measurements and lower vitamin D concentration [16]. In a Korean study it was reported that 65% of men and 91.7% of women with Vitamin D deficiency had BMI over 23 kg/m^2^ (which was the cut-off measurement for overweight among the Asian population) [17]. Our study also found a relationship between Vitamin D deficiency and obesity.

We also revealed that vitamin D level was negatively related to systolic blood pressure measurements. Park et al. also found a significant inverse relationship with serum vitamin D levels and systolic and diastolic blood pressures [18].

In a cross-sectional study, elderly male participants with vitamin D levels < 15.02 ng/mL or < 37.49 nM had a threefold higher prevalence of hypertension compared to those with ≥ 15.02 ng/mL or ≥ 37.49 nM [19].

In a study performed on African Americans, a significant reduction of 0.2 mmHg (*P *= .02) in systolic pressure, but insignificant effect on diastolic pressure (*P *= .37) was reported for each 1-ng/mL increase in plasma of 25-hydroxyvitamin D [20]. It is reported in the US Third National Health and Nutrition Examination Survey (1988–1994) that a higher serum 25(OH)D concentration reduces the risk of diabetes [21]. Some studies also report that supplemental intake of vitamin D and calcium reduces the risk of type 2 diabetes [16, 22]. Our study found an inverse relationship between impaired glucose tolerance and vitamin D level indicating that pre-diabetes were associated with vitamin D deficiency. However, we found that HbA1c was positively associated with vitamin D level. This finding does not correspond with reported studies, which found a relationship between vitamin D deficiency and diabetes. We postulate that participants with known diabetes might already follow a lifestyle modification including diet and physical activity coupled with regular physician visits to monitor HbA1c.

Associations of serum OHVD levels and serum lipids have been reported [18, 22, 23]. In a cross sectional study performed in Norway, a significant increase in total cholesterol, HDL and LDL cholesterol levels were reported increasing across serum of vitamin D quartiles [19]. Our study also reported a high total cholesterol in patients with low vitamin D. Moreover, we reported a significant inverse correlation between vitamin D level and the Framingham risk score (FRS) that corresponded to an American Heart Association’s study, which suggested a moderate-to-severe vitamin D deficiency is a risk factor to develop cardiovascular disease [24].

A 60% higher risk of myocardial infarctions was reported for patients with low vitamin D concentrations (< 15 ng/mL) compared to those with higher vitamin D concentrations in the Framingham Heart Study [24]. A systemic review reported that there could be a reduced CVD risk by using vitamin D supplements at moderate- to- high dosages, whereas calcium supplements had minimal cardiovascular effects. Further Studies are needed to explore the role of both supplements in CVD prevention [25].

### Conclusion

We found a high prevalence of Vitamin D deficiency among the Abu Dhabi national population. The strengths of the study were its large sample size, although the proportion of male (36.9%) vs female (63.1%) representation differed, it was of note that vitamin D deficiency (male 83.1% vs female 83.8%) and insufficiency (male 12.7% vs female 11.2%) was similar for the two gender groups. However, the association of vitamin D deficiency, increased cardiovascular risk, pre-diabetes, and dyslipidemia warrant an additional focus on health promotion and lifestyle intervention not only in the female population, but also in the male population. Given a serious morbidity associated with vitamin D levels, it is essential to manage the vitamin D deficiency appropriately.

## Limitations

Our study had some limitations. Retrospective studies could be subject to recall bias. Secondary causes of vitamin D deficiency e.g., chronic kidney disease were excluded, and there was no reference to abnormal parathormone levels. The electronically generated report for vitamin D treatment was limited to governmental clinics, which excluded private institutions. Although the UAE population consisted of many different nationalities and ethnic groups, our study focused on UAE nationals.

## Data Availability

The data cannot be shared as it owned by the institution (ambulatory health care)

## Abbreviations

25(OH) D: 25-hydroxyvitamin D
BMI: body mass index
SBP: systolic blood pressure
DBP: diastolic Blood pressure
HDL: high-density lipoprotein cholesterol
LDL: low-density lipoprotein cholesterol
BMD: bone mineral density
FRS: Framingham risk score
